# The Meq oncoprotein of Marek's disease virus interacts with p53 and inhibits its transcriptional and apoptotic activities

**DOI:** 10.1186/1743-422X-7-348

**Published:** 2010-11-26

**Authors:** Xufang Deng, Xiangdong Li, Yang Shen, Yafeng Qiu, Zixue Shi, Donghua Shao, Yamei Jin, Hongjun Chen, Chan Ding, Li Li, Puyan Chen, Zhiyong Ma

**Affiliations:** 1 Shanghai Veterinary Research Institute, Chinese Academy of Agricultural Science, Shanghai, 200241, PR China; 2 Guangxi Botanic Garden of Medicinal Plants, Nanning, 530023, PR China; 3 Key Laboratory of Animal Disease Diagnosis and Immunology, Ministry of Agriculture at Nanjing Agricultural University, Nanjing, 210095, PR China

## Abstract

**Background:**

Marek's disease virus (MDV) is an oncogenic herpesvirus, which causes malignant lymphoma in chickens. The Meq protein of MDV, which is expressed abundantly in MDV-infected cells and in Marek's disease (MD) tumor cells, functions as a transcriptional activator and has been proposed to play an important role in oncogenic transformation. Preliminary studies demonstrated that Meq is able to bind p53 *in vitro*, as demonstrated using a protein-binding assay. This observation prompted us to examine whether the interaction between Meq and p53 occurs in cells, and to investigate the biological significance of this interaction.

**Results:**

We confirmed first that Meq interacted directly with p53 using a yeast two-hybrid assay and an immunoprecipitation assay, and we investigated the biological significance of this interaction subsequently. Exogenous expression of Meq resulted in the inhibition of p53-mediated transcriptional activity and apoptosis, as analyzed using a p53 luciferase reporter assay and a TUNEL assay. The inhibitory effect of Meq on transcriptional activity mediated by p53 was dependent on the physical interaction between these two proteins, because a Meq deletion mutant that lacked the p53-binding region lost the ability to inhibit p53-mediated transcriptional activity and apoptosis. The Meq variants L-Meq and S-Meq, but not VS-Meq and ∆Meq, which were expressed in MD tumor cells and MDV-infected cells, exerted an inhibitory effect on p53 transcriptional activity. In addition, ∆Meq was found to act as a negative regulator of Meq.

**Conclusions:**

The Meq oncoprotein interacts directly with p53 and inhibits p53-mediated transcriptional activity and apoptosis. These findings provide valuable insight into the molecular basis for the function of Meq in MDV oncogenesis.

## Background

Marek's disease (MD), which is caused by Marek's disease virus (MDV), is a lymphoproliferative disease of chickens that causes significant economic losses in the poultry industry. MDV belongs to the genus Mardivirus of the Alphaherpesvirinae subfamily, but it shares biological characteristics with gammaherpesviruses, for example its ability to induce T-cell lymphoma and its slow growth in cell culture [1]. MDV replicates in B and T lymphocytes during early cytolytic infection and subsequently establishes a latent infection of T lymphocytes that are finally transformed, which leads to the development of lymphomatous lesions in the visceral organs, peripheral nerves and skin [2]. MD, therefore, serves as an elegant model for understanding the molecular mechanisms of herpesvirus-induced latency and oncogenesis [3].

**Figure 1:**
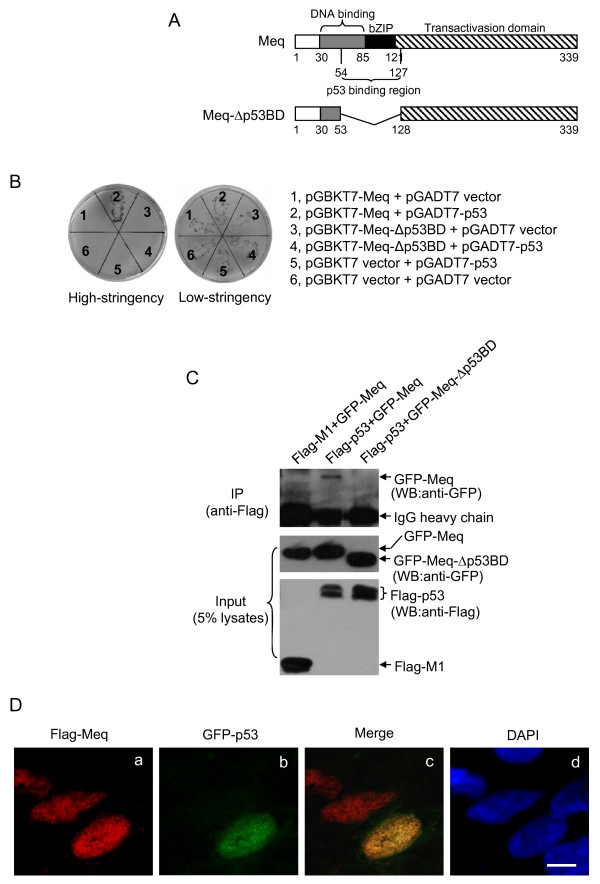
**Interaction between Meq and p53**. (A) Schematic representation of the wild-type Meq protein (Meq) and the Meq protein of the deletion mutant (Meq-Δp53BD), which lacked the p53 binding region. The numbers indicate amino acid positions. (B) Yeast AH109 cells were transformed with a combination of the indicated plasmids and selected on low-stringency and high-stringency media. (C) CEF cells were transfected with a combination of the indicated plasmids and incubated for 24 h. Flag-tagged influenza virus M1 protein (Flag-M1) was used as a negative control. The cell lysates prepared from the transfectants were subjected to immunoprecipitation using anti-Flag antibodies. The immunoprecipitates were immunoblotted with anti-GFP antibodies. The cell lysates were included as a loading control. IP, immunoprecipitation. WB, Western blot. (D) CEF cells were co-transfected with Flag-Meq and GFP-p53 and incubated for 24 h. The transfectants were fixed in a 1:1 solution of methanol/acetone for 20 min at -20°C and immunostained with anti-Flag antibodies (panel a, red). The cells were also stained for DNA with 4',6'-diamidino-2-phenylindole (DAPI) (panel d, blue). Panel c shows the merged images of panels a and b (green). Bar, 5 μm.

The MDV genome encodes at least 80 proteins [4], among which Meq is considered to be the major oncoprotein [3]. Meq is a protein of 339 amino acids (aa) that is expressed during both the cytolytic and the latent/tumor phases of infection [5]. Over-expression of Meq results in transformation of fibroblast cells [6-8]. Furthermore, analysis of a recombinant MDV mutant virus that lacks the *meq *gene demonstrated that Meq is required for transformation of T lymphocytes [9]. Structurally, Meq contains a DNA-binding domain, a basic region-leucine zipper (bZIP) domain that is similar to that of members of the Jun/Fos family of transcriptional activators [10], and a proline-rich transactivation domain at the carboxy terminus [11] (Figure 1A). Like other bZIP proteins, Meq forms homodimers with itself, and heterodimers with cellular proteins that include JunB, c-Jun, c-Fos, SNF, ATF, CREB and C/EBP to transactivate its target genes [3]. In addition, Meq interacts with non-bZIP cellular proteins, such as p53, retinoblastoma protein, cyclin-dependent kinase 2, C-terminal binding protein-1 and heat shock protein 70 [5,12-14]. Despite these observations, the molecular mechanisms of transformation induced by Meq are still not understood completely.

The tumor suppressor protein p53 plays a major role in the protection of cells from malignant transformation via its ability to transactivate target gene expression and mediate downstream events, such as apoptosis and cell cycle arrest [15]. Inhibition of p53-mediated transcriptional activity by viral oncoproteins contributes to virus-mediated oncogenesis. The main mechanism involved is the binding of viral proteins to p53, which reduces its transcriptional activity [16]. For example, SV40 T antigen, adenovirus E1B55K, and HBx from hepatitis B virus bind directly to p53 and inhibit p53-mediated transcriptional activity [17-19]. In the Herpesviridae family, the immediate-early protein BZLF1 and the latency protein EBNA3C of Epstein-Barr virus, a gammaherpesvirus that shares biological characteristics with MDV, have been shown to form a complex with p53 and to disrupt p53-mediated transcriptional activity [20,21]. Given the nature of p53 as a common target for several viral oncoproteins, it is reasonable to speculate that p53 may be a target of the Meq oncoprotein of MDV.

It has been shown previously that p53 has a similar distribution to Meq in MD tumor cells [22], and that Meq is able to bind p53 *in vitro *as demonstrated using a protein-binding assay [12]. These observations prompted us to examine whether the interaction between Meq and p53 occurs in cells, and to investigate the biological significance of this interaction. We found that Meq binds directly to p53 and that this interaction resulted in inhibition of the transcriptional and apoptotic activities of p53.

## Results

### Meq binds directly to p53

The Meq protein has been shown to interact with p53 *in vitro *in a protein-binding assay, and the p53 binding region resides between aa residues 54 and 127 [12] (Figure 1A). To test whether this interaction occurs in cells, we employed a yeast two-hybrid assay. Recombinant plasmids pGBKT7-Meq (wild-type Meq) or pGBKT7-Meq-Δp53BD (a Meq deletion mutant that lacks the p53 binding region), expressing the bait fusion protein, were co-transformed with recombinant plasmid pGADT7-p53 (chicken p53), expressing the prey fusion protein, into yeast AH109 cells and selected on low-stringency and high-stringency media. The yeast cells co-transformed with the vectors pGBKT7-Meq and pGADT7 did not grow on high-stringency medium (Figure 1B, section 1), which suggests that Meq did not activate reporter genes autonomously. However, when pGBKT7-Meq was co-transformed with pGADT7-p53, the yeast cells grew on high-stringency medium (Figure 1B, section 2), which suggests that Meq interacted with p53. The yeast cells co-transformed with pGBKT7-Meq-Δp53BD and pGADT7-p53 did not grow on high-stringency medium (Figure 1B, section 4), confirming that the region of the Meq protein that spans aa residues 54 to 127 is required for the interaction with p53.

To determine whether the interaction between Meq and p53 occurs in host cells naturally permissive for MDV, primary chick embryo fibroblasts (CEFs) were co-transfected with Flag-tagged chicken p53 (Flag-p53) and GFP-tagged Meq (GFP-Meq) or GFP-tagged Meq-Δp53BD (GFP-Meq-Δp53BD) and analyzed by an immunoprecipitation assay. The Flag-tagged influenza virus M1 protein (Flag-M1) was used as a negative control. CEFs were used in this assay for the following reasons: (i) they are naturally permissive for MDV replication, and (ii) they can be transformed by MDV [6]. Flag-p53 immunoprecipitated GFP-Meq, but not GFP-Meq-Δp53BD (Figure 1C), which confirms that the interaction between Meq and p53 occurs in host cells that are naturally permissive for MDV.

It has been reported previously that the subcellular localization of p53 is similar to that of Meq in MD tumor cells [22]. Given that there is no commercial antibody suitable for the detection of chicken p53, we visualized the subcellular localization of Meq and p53 in CEFs that were co-transfected transiently with GFP-p53 and Flag-tagged Meq (Flag-Meq). The Flag-Meq protein was expressed in the nucleus (Figure 1D, panel a), as reported previously [23]. The co-localization of Flag-Meq and GFP-p53 was observed in CEFs (Figure 1D, panel c), and also in other types of cells, such as H1299, DF-1 and Vero cells (data not shown).

### Meq inhibits the transcriptional activity of p53

**Figure 2:**
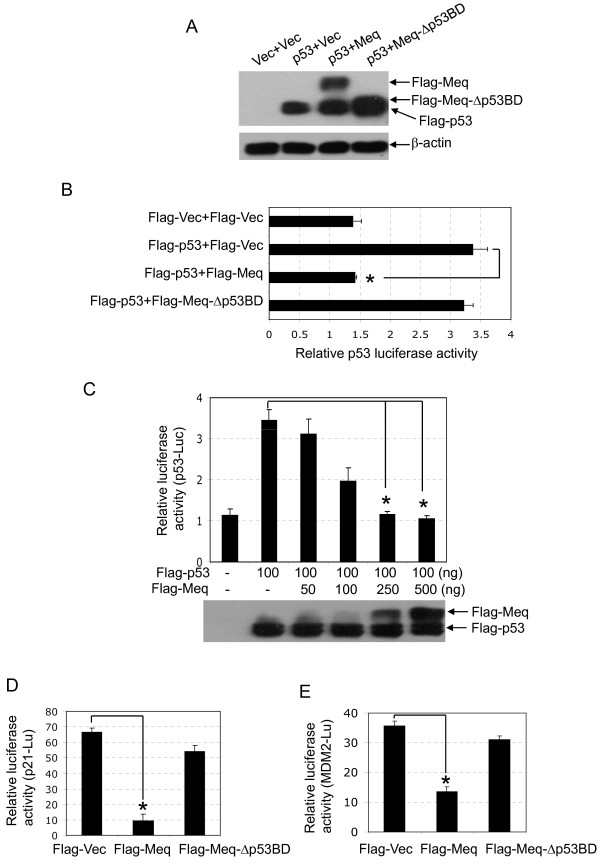
**Meq inhibits the transcriptional activity of p53**. (A and B) H1299 cells were co-transfected transiently with a combination of the indicated plasmids in the presence of the p53 luciferase reporter plasmid (p53-Luc). The expression of the indicated plasmids was detected by western blot analysis (A). The luciferase activity of the transfectants was measured 24 h post-transfection (B). *p < 0.05 compared with cells transfected with Flag-p53 alone (Flag-p53+Flag-Vec). (C) H1299 cells were transfected transiently with increasing amounts of Flag-Meq in the presence of Flag-p53 and p53-Luc. The expression of Flag-Meq and Flag-p53 was detected by western blot analysis. *p < 0.05 compared with cells transfected with Flag-p53 alone (Flag-p53+Flag-Vec). (D and E) CEF cells were transfected transiently with the indicated plasmids in the presence of p21 promoter luciferase reporter plasmids (D) or MDM2 promoter luciferase reporter plasmids (E). *p < 0.05 compared with cells transfected with Flag-vector (Flag-Vec).

The transcriptional activity of p53 is important for p53-mediated regulation [15], and most viral proteins that interact with p53 have been reported to suppress p53 transcriptional activity [16]. Therefore, to investigate whether the interaction between Meq and p53 influences the transcriptional activity of p53, p53-null H1299 cells were co-transfected with Flag-p53 and Flag-Meq in the presence of a chicken p53 luciferase reporter plasmid (p53-Luc) that contains four tandem repeats of the chicken p53 consensus binding site [24]. The luciferase activity was measured 24 h post-transfection. The expression of Flag-Meq and Flag-p53 in the transfectants was confirmed by western blot analysis (Figure 2A). Expression of Flag-p53 alone (Flag-p53+Flag-Vec) resulted in a significant enhancement of luciferase activity when compared with the Flag-vector (Flag-Vec+Flag-Vec), but the enhanced luciferase activity was reduced significantly by co-expression of Flag-Meq (Flag-p53+Flag-Meq) (Figure 2B). Next, H1299 cells were co-transfected with Flag-p53 and increasing amounts of Flag-Meq in the presence of p53-Luc. The expression of Flag-Meq and Flag-p53 was detected by western blot analysis. The luciferase activity of the transfectants decreased gradually with increasing expression of Flag-Meq in a dose-dependent manner (Figure 2C). These results suggested that Meq inhibited the transcriptional activity of p53.

To assess the inhibitory effect of Meq on the transcriptional activity of p53 further, we analyzed the influence of Meq on the expression of the p53 targeting genes p21 [25] and MDM2 [26] in CEFs using a luciferase assay. A p21 promoter luciferase reporter plasmid (p21-Luc) and an MDM2 promoter luciferase reporter plasmid, both containing p53 response elements, were co-transfected separately with Flag-Meq into CEFs, and the luciferase activities were measured 24 h post-transfection. As shown in Figure 2D and 2E, Meq reduced the luciferase activity of both p21-Luc and MDM2-Luc significantly, when compared with the Flag-vector (Flag-Vec). Taken together, these observations suggested that Meq inhibits p53-mediated transcriptional activity.

### p53-mediated apoptosis is suppressed by Meq expression

**Figure 3:**
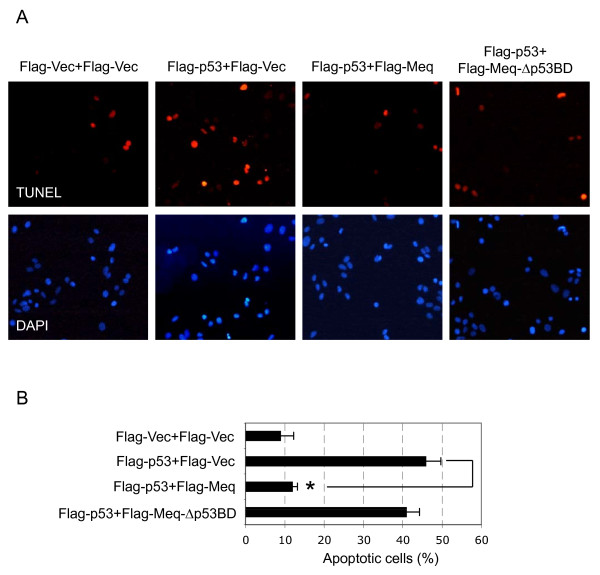
**Meq inhibits p53-mediated apoptosis**. Flag-p53 was co-transfected into CEFs with Flag-Meq or Flag-Meq-Δp53BD at a 1:10 molar ratio. (A) Apoptotic cells (red) were stained with a TUNEL assay kit. The cells were also stained for DNA with DAPI (blue). (B) The percentage of apoptotic cells (red) was determined and is shown on the graph as the average ± standard error from three experiments. More than 500 cells were examined for each experiment. *p < 0.05 compared with cells transfected with Flag-p53 alone (Flag-p53+Flag-Vec).

To explore the functional significance of the interaction between Meq and p53, we determined whether Meq affects p53-mediated apoptosis, an active physiological response that eliminates mutated or preneoplastic cells. Flag-Meq was co-transfected with Flag-p53 into CEFs, and apoptosis of the transfectants was analyzed using a TUNEL assay. The expression of Flag-p53 and Flag-Meq in the transfectants was confirmed by western blot analysis (data not shown). As shown in Figure 3, apoptosis was detected in approximately 46% of cells transfected with Flag-p53 alone (Flag-p53+Flag-Vec), which was significantly higher than the percentage of apoptotic cells among those transfected with the Flag-vector (Flag-Vec+Flag-Vec). This suggests that Flag-p53 was able to induce apoptosis in CEFs. However, in the presence of Flag-Meq, p53-mediated apoptosis was reduced dramatically, and was observed in only 12% of cells (Flag-p53+Flag-Meq). These data revealed that Meq inhibited p53-mediated apoptosis.

### Meq inhibits p53 transcriptional activity, dependent on physical interaction

A number of tumor virus proteins, such as SV40 T antigen, E1B55K and HBx, disrupt the function of p53, and this inhibition is dependent on the physical interaction between these proteins [17-19]. We explored, therefore, whether the inhibitory effect of Meq on p53 transcription was dependent on their physical interaction. Flag-p53 was co-transfected with Flag-Meq or Flag-Meq-Δp53BD into H1299 cells in the presence of p53-Luc. The deletion mutant of Meq-Δp53BD was unable to bind p53, as shown in Figure 1. The inhibitory effects of Flag-Meq and Flag-Meq-Δp53BD on the luciferase activity of p53-Luc were compared subsequently. The expression of Flag-Meq reduced the luciferase activity significantly; however, in contrast, no inhibitory effect on luciferase activity was observed in the cells transfected with Flag-Meq-Δp53BD (Figure 2A and 2B). Similar results were observed in experiments that compared the inhibitory effects of Flag-Meq and Flag-Meq-Δp53BD on the luciferase activities of p21-luc and MDM2-Luc (Figure 2D and 2E). Next, we compared the inhibitory effects of Flag-Meq and Flag-Meq-Δp53BD on apoptosis mediated by p53. As expected, Flag-Meq-Δp53BD did not inhibit p53-mediated apoptosis (Figure 3). Taken together, these data suggested that Meq inhibits p53 transcription and that this inhibition is dependent on the physical interaction between Meq and p53.

### Effects of Meq variants on p53 transcriptional activity

**Figure 4:**
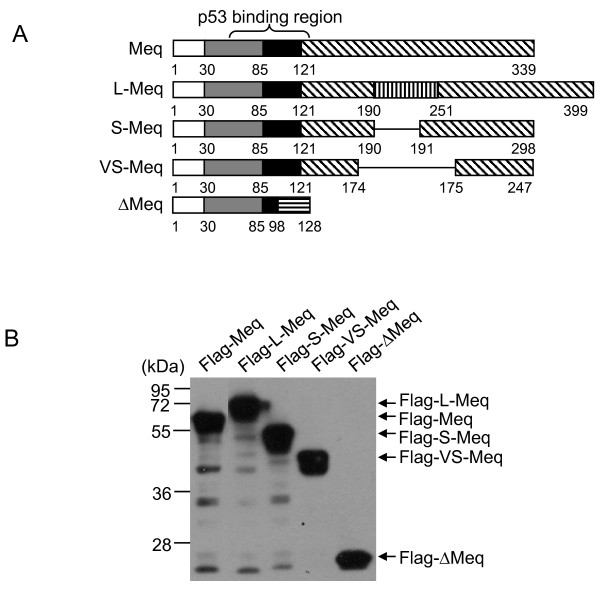
**Construction of Meq variants**. (A) Schematic representation of Meq variants (L-Meq, S-Meq, VS-Meq and ∆Meq). The numbers indicate amino acid positions (please refer to Fig. 1A for the protein structure of Meq). (B) H1299 cells were transfected transiently with each Flag-tagged Meq variant and the expression was determined by western blot analysis 24 h post-transfection.

**Figure 5:**
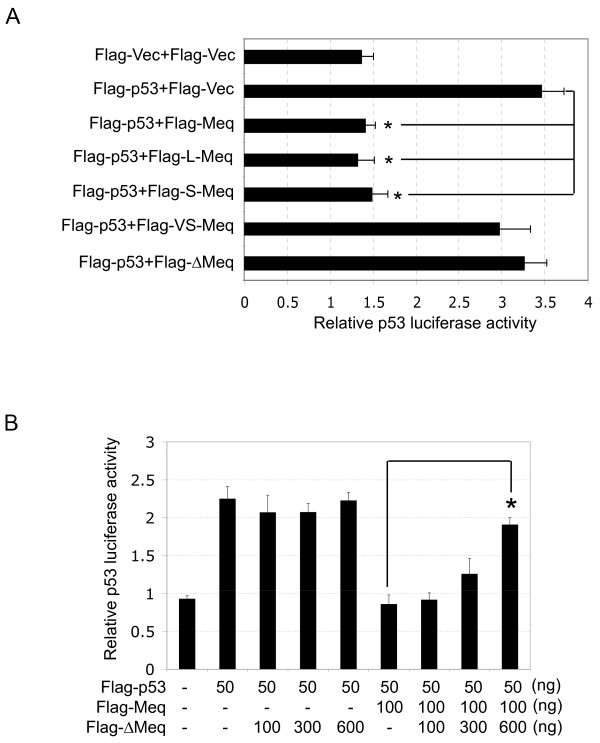
**Analysis of the effect of the Meq variants on p53 transcriptional activity**. (A) H1299 cells were co-transfected transiently with a combination of the indicated plasmids in the presence of the p53 luciferase reporter plasmid. *p < 0.05 compared with cells transfected with Flag-p53 alone (Flag-p53+Flag-Vec). (B) H1299 cells were co-transfected transiently with a combination of the indicated plasmids in the presence of p53 luciferase reporter plasmid. *p < 0.05 compared with cells transfected with Flag-Meq alone.

Meq is a polymorphic protein and several variants have been characterized, including L-Meq (which contains an insertion of 60 aa between residues190 and 191), S-Meq (which contains a deletion of 41 aa between residues 190 and 191), VS-Meq (which contains a deletion of 92 aa between residues 174 and 175), and ∆Meq (composed of 98 aa from the N-terminal region of Meq and a frame-shifted distinct C-terminus of 30 aa) [27-29] (Figure 4A). Meq and its variants are expressed in MD tumor cells and in MDV-infected cells, but their roles in cytolytic infection and the establishment of latency or transformation have not been elucidated fully. We constructed recombinant plasmids that expressed Flag-tagged Meq variants and confirmed their expression in transfected cells by western blot analysis (Figure 4B). To investigate the effects of the Meq variants on p53 transcriptional activity, we co-transfected each Meq variant with Flag-p53 into H1299 cells and analyzed p53 transcriptional activity using a luciferase assay. As shown in Figure 5A, L-Meq and S-Meq inhibited p53 transcriptional activity significantly, with a similar efficiency to Meq, while ∆Meq showed no detectable inhibitory effect on transcription of p53. Interestingly, VS-Meq, which contains the p53 binding region but lacks a 92-aa region of the transactivation domain, showed no inhibitory effect on p53 transcriptional activity.

It has been reported previously that L-Meq and ∆Meq are negative regulators of Meq, and suppress the transactivational activities of Meq [28,29]. It was therefore of interest to investigate the effects of the Meq variants on the p53-inhibitory activity of Meq. Flag-Meq was co-transfected with increasing amounts of each Meq variant into H1299 cells in the presence of Flag-p53 and p53-Luc, and the luciferase activities were measured 24 h post-transfection. L-Meq and S-Meq showed no enhanced or suppressive effect, while VS-Meq showed a slight, but not significant, inhibitory effect on the p53-inhibitory activity of Meq (data not shown). In contrast, the inhibitory effect of Meq on the transcriptional activity of p53 was attenuated significantly by co-expression of ∆Meq, in a dose-dependent manner (Figure 5B), which suggests that ∆Meq is a negative regulator of Meq. Taken together, these data suggested that the variants of Meq have different effects on the transcriptional activity of p53, and may play different roles during cytolytic infection and the establishment of latency or transformation.

## Discussion

Herpesviruses are important pathogens that are associated with a wide range of diseases in humans and other animals. MDV is one of the most contagious and highly oncogenic herpesviruses, and MD is the only neoplastic disease for which an effective vaccine has been employed widely [3]. However, with increasing reports of vaccination breaks and the emergence of more virulent pathotypes, MD continues to pose a severe threat to the poultry industry, and the development of more effective control strategies remains a significant challenge [4]. Therefore, a fundamental understanding of the molecular mechanisms of MD oncogenesis is important, not only for the development of more sustainable control strategies, but also to increase understanding of some of the principles of virus-induced lymphomagenesis.

The tumor suppressor protein p53 plays a major role in the protection of cells from malignant transformation and has been targeted by numerous viral oncoproteins [16]. A preliminary study reported that Meq binds to p53 *in vitro*, as determined by a protein-binding assay [12]. In this study, we used a yeast two-hybrid assay to show that Meq interacts directly with p53 (Figure 1B). We also demonstrated the interaction between Meq and p53 in host cells naturally permissive for MDV (Figure 1C), which confirmed further the interaction between these two proteins. Given that the tumor suppressor function of p53 is linked closely to its ability to transactivate target gene expression and mediate downstream events [15], we investigated the biological significance of the interaction between Meq and p53 on p53-mediated transcriptional activities. Exogenous expression of Meq resulted in inhibition of p53-mediated transcriptional activity and apoptosis (Figures 2 and 3), which suggests that p53 is targeted by the Meq oncoprotein of MDV.

Although the mechanisms of the abrogation of p53 transcriptional activity in virus-induced oncogenesis are not understood fully, the main mechanism employed by oncoviruses involves the direct binding of viral proteins to p53. This results in modulation of the functions of p53, mainly via acceleration of its degradation, sequestration of p53 in the cytoplasm, blockage of the DNA-binding capacity of p53, and/or blockage of the interaction of p53 with transcription coactivators [16]. We found that Meq-Δp53BD, the Meq deletion mutant that lacks the p53 binding region and is unable to bind p53 (Figure 1), did not inhibit p53-mediated transcriptional activity and apoptosis (Figures 2 and 3). This suggests that the inhibitory effect of Meq on the transcription of p53 is dependent on the physical interaction of these two proteins. However, this interaction between Meq and p53 did not affect the stability of the protein or the subcellular localization of p53 (data not shown). The mechanism that underlies the p53-inhibitory effect of Meq is a current topic of investigation in our laboratory.

Several variants of Meq, including L-Meq, S-Meq, VS-Meq and ∆Meq (Figure 4A), have been characterized in MD tumor cells and MDV-infected cells [27-29], but their functions during cytolytic infection and the establishment of latency or transformation have not been elucidated fully. In the context of the inhibition of the function of p53, L-Meq and S-Meq were found to inhibit p53 transcriptional activity with a similar efficiency to Meq (Figure 5A). This implies that L-Meq and S-Meq may also play a role in cellular transformation, in a similar way to the Meq protein. Interestingly, VS-Meq, which contains the p53-binding region but lacks a 92-aa region located in the transactivation domain, showed no inhibitory effect on the transcriptional activity of p53 (Figure 5A), which suggests that Meq requires additional region(s) to exert this inhibitory function cooperatively. Although ∆Meq did not show a significant inhibitory effect on the transcriptional activity of p53, it suppressed the p53-inhibitory activity of Meq significantly (Figure 5B), which suggests that it acts as a negative regulator of Meq, as demonstrated in a previous study [29]. These data also suggested that Meq proteins play complex roles during cytolytic infection and the establishment of latency or transformation.

## Conclusions

In conclusion, we confirmed that Meq interacted directly with p53. Exogenous expression of Meq resulted in the inhibition of p53-mediated transcriptional activity and apoptosis. The inhibitory effect of Meq on p53-mediated transcriptional activity was dependent on the physical interaction between these two proteins. The Meq variants L-Meq and S-Meq, but not VS-Meq and ∆Meq, exerted inhibitory effects on the transcriptional activity of p53. In addition, ∆Meq was found to work as a negative regulator of Meq. Our findings provide valuable insight into the molecular basis of the function of Meq in the oncogenesis of MDV.

## Methods

### Cells, viruses and antibodies

The CEFs were prepared from nine-day-old embryonated specific-pathogen-free chicken eggs and cultured using standard techniques. The human non-small lung cancer cell line H1299 (p53-null) and the MD tumor cell line MSB-1 were maintained in Dulbecco's modified Eagle's medium and RPMI 1640 medium, respectively, supplemented with 10% fetal bovine serum, in an atmosphere containing 5% CO_2_. A very virulent strain of MDV, strain RB1B, was propagated on CEFs. The commercial antibodies used were an anti-Flag monoclonal antibody (M2, Sigma, St. Louis, MO, USA), a rabbit anti-Flag polyclonal antibody (Sigma), an anti-GFP monoclonal antibody (ab1218, Abcam, Cambridge, MA, USA), an anti-β-actin monoclonal antibody (AC-15, Sigma), a horseradish peroxidase (HRP)-conjugated goat anti-rabbit IgG antibody (sc-2004, Santa Cruz Biotechnology, Santa Cruz, CA, USA), a HRP-conjugated goat anti-mouse IgG antibody (sc-2005, Santa Cruz) and an Alexa Fluor 594-conjugated goat anti-mouse IgG (H+L)2 monoclonal antibody (Molecular Probes, Eugene, OR, USA).

### Construction of expression plasmids and transient transfection

The full-length DNA fragment encoding the wild-type Meq protein was amplified by PCR from the RB1B MDV strain and subcloned into the expression vectors p3xFLAG-CMV-7.1 and pEGFP-C1, to generate the recombinant plasmids Flag-Meq and GFP-Meq, respectively. The L-Meq variant was amplified by PCR from MSB-1 cells and subcloned into the expression vector p3xFLAG-CMV-7.1. The Meq deletion mutant (Meq-Δp53BD) that lacked the p53 binding domain (Figure 1A), and several variants of Meq including S-Meq, VS-Meq and ∆Meq (Figure 4A), were generated by PCR-based site-directed mutagenesis [30] using Flag-Meq as the template. The primers used are shown in Table 1. Chicken p53 cDNA [31], a gift provided generously by Dr. Thierry Soussi from Université Pierre et Marie Curie-Paris, France, was subcloned into the expression vectors p3xFLAG-CMV-7.1 and pEGFP-C1 to generate the recombinant plasmids Flag-p53 and GFP-p53, respectively. The cells were plated onto tissue culture plates 24 h before transfection. Transfection was performed using the Lipofectamine2000 transfection reagent (Invitrogen, Carlsbad, CA, USA) according to the manufacturer's instructions.

**Table 1:** primer sequence

Gene name	Primer sequence (5' to 3')
Meq	GCGAATTCTATGTCTCAGGAGCCAGAGCC
	TTATCTCGAGTCAGGGTCTCCCGTCACC
Meq-Δp53BD	CCTTCCCTGACGGCCTATCTGTACCCCTAACGGTGACCCT
	AGGGTCACCGTTAGGGGTACAGATAGGCCGTCAGGGAAGG
L-Meq	GCGAATTCTATGTCTCAGGAGCCAGAGCC
	GGCTCGAGTTATGAGGGCGCAAACTT
S-Meq	GCGCCCAGCTCTGCTCGACCCCACCACCTCCCATCTGTAC
	GTACAGATGGGAGGTGGTGGGGTCGAGCAGAGCTGGGCGC
VS-Meq	CCCAACCTCCTATCTGTACCCCTCCATCGCCGGGGACGGT
	ACCGTCCCCGGCGATGGAGGGGTACAGATAGGAGGTTGGG
∆Meq	GCTGCAGAGGGCCAATGAACACCGAGGATCCCGAACAGGA
	TCCTGTTCGGGATCCTCGGTGTTCATTGGCCCTCTGCAGC

### Luciferase assay

Cells were transfected with the indicated expression plasmids in the presence of the luciferase reporter plasmid and the control plasmid, Renilla luciferase pRL-TK (Promega, Madison, WI, USA). The chicken p53 luciferase reporter plasmid (p53-Luc) was provided generously by Dr. Byung-Whi Kong from the University of Arkansas, USA [24]. The p21 luciferase reporter plasmid (p21-Luc) and MDM2 luciferase reporter plasmid (MDM2-Luc) were gifts from Dr. Kenji Fukasawa (H. Lee Moffitt Cancer Center & Research Institute, USA). Transfectants were harvested 24 h post-transfection and luciferase assays were carried out with the Dual-luciferase reporter assay system (Promega), according to the manufacturer's protocol. The firefly luciferase activity of individual cell lysates was normalized to Renilla luciferase activity. All assays were performed at least in triplicate.

### TUNEL assay

CEFs grown on coverslips were co-transfected transiently with Flag-p53 and Flag-Meq or Flag-Meq-Δp53BD at a molar ratio of 1:10 and incubated for 24 h.

The transfectants were fixed with 4% paraformaldehyde, stained using the In Situ Cell Death Detection Kit, TMR red (Roche, Mannheim, Germany), and examined under a fluorescence microscope.

### Yeast two-hybrid assay

The yeast two-hybrid assay was carried out using the MATCHMAKER GAL4 two-hybrid system 3 (Clontech, Palo Alto, CA, USA), and all procedures were performed according to the manufacturer's protocols. Meq and Meq-Δp53BD were subcloned in-frame with the GAL4 DNA binding domain into the pGBKT7 vector to generate the bait plasmids pGBKT7-Meq and pGBKT7-Meq-Δp53BD, respectively. Cells of the yeast host strain AH109 were transformed with pGBKT7-Meq or pGBKT7-Meq-Δp53BD to confirm that they did not activate reporter genes autonomously. Chicken p53 was subcloned in-frame with the GAL4 activation domain into the pGADT7 vector to generate the prey plasmid pGADT7-p53. To investigate the interaction between p53 and Meq, pGADT7-p53 was co-transformed with pGBKT7-Meq or pGBKT7-Meq-Δp53BD into yeast AH109 cells. The transformants were selected on low-stringency medium plates lacking tryptophan and leucine, and on high-stringency medium plates lacking tryptophan, leucine, histidine and adenine. The positive clones were confirmed by PCR analysis.

### Western blot analysis, immunofluorescence and immunoprecipitation assays

Western blot analysis, immunofluorescence and immunoprecipitation assays were performed as described previously [32].

### Statistics

All measured values are expressed as the mean ± SE. The significance of the results was analyzed using Student's *t*-test, and *p *values less than 0.05 were considered significant.

## Competing interests

The authors declare that they have no competing interests.

## Authors' contributions

XFD and XDL carried out most of the experiments and wrote the manuscript. YS and YFQ constructed the experimental plasmids. ZXS and DHS helped with the experiments. YMJ advised and helped in yeast two-hybrid assay. HJC and CD cultured and maintained CEF and MSB-1 cells. LL and PYC revised the experimental design. ZYM designed the experiments and revised the manuscript. All of the authors read and approved the final version of this manuscript.

## References

[B1] Ross NL T-cell transformation by Marek's disease virus Trends Microbiol 1999 7 22 29 10.1016/S0966-842X(98)01427-9 10068994

[B2] Calnek BW Pathogenesis of Marek's disease virus infection Curr Top Microbiol Immunol 2001 255 25 55 11217426 10.1007/978-3-642-56863-3_2

[B3] Osterrieder N Kamil JP Schumacher D Tischer BK Trapp S Marek's disease virus: from miasma to model Nat Rev Microbiol 2006 4 283 294 10.1038/nrmicro1382 16541136

[B4] Venugopal K Marek's disease: an update on oncogenic mechanisms and control Res Vet Sci 2000 69 17 23 10.1053/rvsc.2000.0396 10924389

[B5] Liu JL Ye Y Qian Z Qian Y Templeton DJ Lee LF Kung HJ Functional interactions between herpesvirus oncoprotein MEQ and cell cycle regulator CDK2 J Virol 1999 73 4208 4219 10196317 10.1128/jvi.73.5.4208-4219.1999 PMC104200

[B6] Buranathai C Rodríguez J Grose C Transformation of primary chick embryo fibroblasts by Marek's disease virus Virology 1997 239 20 35 10.1006/viro.1997.8854 9426443

[B7] Liu JL Ye Y Lee LF Kung HJ Transforming potential of the herpesvirus oncoprotein MEQ: morphological transformation, serum-independent growth, and inhibition of apoptosis J Virol 1998 72 388 395 9420237 10.1128/jvi.72.1.388-395.1998 PMC109386

[B8] Levy AM Gilad O Xia L Izumiya Y Choi J Tsalenko A Yakhini Z Witter R Lee L Cardona CJ Kung HJ Marek's disease virus Meq transforms chicken cells via the v-Jun transcriptional cascade: a converging transforming pathway for avian oncoviruses Proc Natl Acad Sci USA 2005 102 14831 14836 10.1073/pnas.0506849102 16203997 PMC1253582

[B9] Lupiani B Lee LF Cui X Gimeno I Anderson A Morgan RW Silva RF Witter RL Kung HJ Reddy SM Marek's disease virus-encoded Meq gene is involved in transformation of lymphocytes but is dispensable for replication Proc Natl Acad Sci USA 2004 101 11815 11820 10.1073/pnas.0404508101 15289599 PMC511057

[B10] Jones D Lee L Liu JL Kung HJ Tillotson JK Marek's disease virus encodes a basic-leucine zipper gene resembling the fos/jun oncogenes that is highly expressed in lymphoblastoid tumors Proc Natl Acad Sci USA 1992 89 4042 4046 10.1073/pnas.89.9.4042 1315048 PMC525628

[B11] Anobile JM Arumugaswami V Downs D Czymmek K Parcells M Schmidt CJ Nuclear localization and dynamic properties of the Marek's disease virus oncogene products Meq and Meq/vIL8 J Virol 2006 80 1160 1166 10.1128/JVI.80.3.1160-1166.2006 16414993 PMC1346918

[B12] Brunovskis P Qian Z Li D In The 5th International Symposium on Marek's disease 1996 Kellogg Center, Michigan State University, East Lansing, Michigan: AAAP, Kennett Square, Pennsylvania 265 270

[B13] Brown AC Baigent SJ Smith LP Chattoo JP Petherbridge LJ Hawes P Allday MJ Nair V Interaction of MEQ protein and C-terminal-binding protein is critical for induction of lymphomas by Marek's disease virus Proc Natl Acad Sci USA 2006 103 1687 1692 10.1073/pnas.0507595103 16446447 PMC1413633

[B14] Zhao Y Kurian D Xu H Petherbridge L Smith LP Hunt L Nair V Interaction of Marek's disease virus oncoprotein Meq with heat shock protein 70 in lymphoid tumour cells J Gen Virol 2009 90 2201 2208 10.1099/vir.0.012062-0 19494050

[B15] Levine AJ p53, the cellular gatekeeper for growth and division Cell 1997 88 323 331 10.1016/S0092-8674(00)81871-1 9039259

[B16] Collot-Teixeira S Bass J Denis F Ranger-Rogez S Human tumor suppressor p53 and DNA viruses Rev Med Virol 2004 14 301 319 10.1002/rmv.431 15334538

[B17] Yew PR Berk AJ Inhibition of p53 transactivation required for transformation by adenovirus early 1B protein Nature 1992 357 82 85 10.1038/357082a0 1533443

[B18] Elmore LW Hancock AR Chang SF Wang XW Chang S Callahan CP Geller DA Will H Harris CC Hepatitis B virus X protein and p53 tumor suppressor interactions in the modulation of apoptosis Proc Natl Acad Sci USA 1997 94 14707 14712 10.1073/pnas.94.26.14707 9405677 PMC25100

[B19] Sheppard HM Corneillie SI Espiritu C Gatti A Liu X New insights into the mechanism of inhibition of p53 by simian virus 40 large T antigen Mol Cell Biol 1999 19 2746 2753 10082540 10.1128/mcb.19.4.2746 PMC84067

[B20] Sato Y Shirata N Kudoh A Iwahori S Nakayama S Murata T Isomura H Nishiyama Y Tsurumi T Expression of Epstein-Barr virus BZLF1 immediate-early protein induces p53 degradation independent of MDM2, leading to repression of p53-mediated transcription Virology 2009 388 204 211 10.1016/j.virol.2009.03.017 19375142

[B21] Yi F Saha A Murakami M Kumar P Knight JS Cai Q Choudhuri T Robertson ES Epstein-Barr virus nuclear antigen 3C targets p53 and modulates its transcriptional and apoptotic activities Virology 2009 388 236 247 10.1016/j.virol.2009.03.027 19394062 PMC4287381

[B22] Gimeno IM Witter RL Fadly AM Silva RF Novel criteria for the diagnosis of Marek's disease virus-induced lymphomas Avian Pathol 2005 34 332 340 10.1080/03079450500179715 16147570

[B23] Liu JL Lee LF Ye Y Qian Z Kung HJ Nucleolar and nuclear localization properties of a herpesvirus bZIP oncoprotein, MEQ J Virol 1997 71 3188 3196 9060682 10.1128/jvi.71.4.3188-3196.1997 PMC191451

[B24] Kim H You S Kim IJ Foster LK Farris J Ambady S Ponce de León FA Foster DN Alterations in p53 and E2F-1 function common to immortalized chicken embryo fibroblasts Oncogene 2001 20 2671 2682 10.1038/sj.onc.1204378 11420679

[B25] Tang HY Zhao K Pizzolato JF Fonarev M Langer JC Manfredi JJ Constitutive expression of the cyclin-dependent kinase inhibitor p21 is transcriptionally regulated by the tumor suppressor protein p53 J Biol Chem 1998 273 29156 29163 10.1074/jbc.273.44.29156 9786925

[B26] Juven T Barak Y Zauberman A George DL Oren M Wild type p53 can mediate sequence-specific transactivation of an internal promoter within the mdm2 gene Oncogene 1993 8 3411 3416 8247544

[B27] Chang KS Ohashi K Onuma M Suppression of transcription activity of the MEQ protein of oncogenic Marek's disease virus serotype 1 (MDV1) by L-MEQ of non-oncogenic MDV1 J Vet Med Sci 2002 64 1091 1095 10.1292/jvms.64.1091 12520099

[B28] Chang KS Ohashi K Onuma M Diversity (polymorphism) of the meq gene in the attenuated Marek's disease virus (MDV) serotype 1 and MDV-transformed cell lines J Vet Med Sci 2002 64 1097 1101 10.1292/jvms.64.1097 12520100

[B29] Okada T Takagi M Murata S Onuma M Ohashi K Identification and characterization of a novel spliced form of the meq transcript in lymphoblastoid cell lines derived from Marek's disease tumours J Gen Virol 2007 88 2111 2120 10.1099/vir.0.82744-0 17622612

[B30] Li X Qiu Y Shen Y Ding C Liu P Zhou J Ma Z Splicing together different regions of a gene by modified polymerase chain reaction-based site-directed mutagenesis Anal Biochem 2008 373 398 400 10.1016/j.ab.2007.10.021 18022376

[B31] Soussi T Bègue A Kress M Stehelin D May P Nucleotide sequence of a cDNA encoding the chicken p53 nuclear oncoprotein Nucleic Acids Res 1988 16 11383 10.1093/nar/16.23.11383 3060861 PMC339033

[B32] Qiu Y Shen Y Li X Liu Q Ma Z Polyclonal antibody to porcine p53 protein: a new tool for studying the p53 pathway in a porcine model Biochem Biophys Res Commun 2008 377 151 155 10.1016/j.bbrc.2008.09.117 18840405

